# Evaluation and Management of Sacroiliac Dysfunction Utilizing an Evidence-Based Algorithmic Approach: A Case Study

**DOI:** 10.7759/cureus.9907

**Published:** 2020-08-20

**Authors:** David P Newman, Brian C McLean, Alexandra M Scozzafava

**Affiliations:** 1 Pain Management-Physiotherapy, Interdisciplinary Pain Management Center, Tripler Army Medical Center, Honolulu, USA; 2 Anesthesiology, Interdisciplinary Pain Management Center, Tripler Army Medical Center, Honolulu, USA; 3 Physical Therapy, Irwin Army Community Hospital, Manhattan, USA

**Keywords:** sacroiliac joint, si, sij, back pain, manipulation, sacroiliac, low back pain

## Abstract

The sacroiliac joint (SIJ) is an important contributor to persistent and functionally limiting lower back pain. Despite extensive debate and research, there is no definitive treatment recommendation or high-level evidence to support a conservative care treatment approach, nor interventional or surgical management procedures for the alleviation of pain originating from the SIJ. Traditional physical therapy and conservative approaches to generalized lower back pain often fail in this patient subset prompting sub-specialty consultation to a pain management center. Diagnosis of the SIJ as the pain generator can be accomplished through physical exam maneuvers and comparative diagnostic blocks; however, upon diagnosis, management remains a challenge. After the diagnosis of SIJ dysfunction is made in our young and active patient population, we have seen significant success in the application of an interdisciplinary and evidence-based treatment algorithm similar to the presented case. To our knowledge, this treatment approach has not been previously described.

## Introduction

Sacroiliac joint (SIJ) dysfunction is a common source of low back pain [1]. The prevalence of SIJ pain is approximately 25% (ranging from 10% to 62% based on setting) in patients with mechanical low back pain below the level of L5 [2]. The location of pain upon presentation can be unilateral or bilateral but is most often not midline [3]. Females are more likely to present with SIJ dysfunction than males [4].

The etiology of SIJ pain is not well understood or agreed upon. While the SIJ itself may be the primary source of pain, mechanical dysfunction at the joint or within the surrounding structures can alter the load-transfer function at the SIJ complex, thereby producing a painful stimulus [1]. SIJ pain can be associated with several inflammatory conditions to include osteoarthritis, inflammatory arthritis, ankylosing spondylitis, infectious and post-traumatic arthritis [5]. Similarly, mechanical faults at the pubic symphysis or SIJ can result in pelvic asymmetry or joint instability [5]. SIJ stiffness, joint hypermobility, and insufficient pelvic girdle stability result in faulty load transfer to the spine or lower extremity and increased shear forces through the SIJ [6].

Managing patients with SIJ dysfunction is difficult due to the multifactorial causes of pain and poor diagnostic clarity during workup. There is low correlation between patient history and diagnosis, limited diagnostic accuracy with imaging [2], and poor validity and reliability of single provocation or special tests during physical exam [7]. Diagnostic clarity may be achieved through an SIJ double anesthetic block; however, this requires consultation with an interventional specialist. The purpose of this case study was to describe the application of a novel evaluation and management algorithm for the treatment of a patient with SIJ dysfunction.

## Case presentation

A 25-year-old male, active duty U.S. Marine, presented with a three-year history of left-sided lower back pain and intermittent radiating pain down the left posterior thigh to the back of the knee. The initial onset of pain was attributed to squatting 135 pounds as part of his training program as a casket bearer for the Marine Corps. He reported that while squatting, his left side “gave away” and he immediately experienced radiating pain down his posterior left leg but was otherwise neurologically intact. The MRI report of his lumbar spine performed at an outside institution noted mild L2-S1 disc degeneration with no canal or foraminal stenosis. The patient underwent an extensive multimodal treatment program to address presumptive discal pathology consisting of physical therapy and chiropractics over a two-year period. He reported minimal improvement with treatment and kept up with his work activities despite the pain. He experienced an acute flare in pain approximately one year prior to presentation to our clinic while lifting a 45-pound plate. This pain recurrence prompted him to re-initiate physical therapy where he was prescribed a program of core strengthening without significant benefit. He was subsequently referred to the Interdisciplinary Pain Management Clinic (IPMC) at Tripler Army Medical Center (TAMC), Honolulu, Hawaii for evaluation and management. At presentation he was 72 inches, 215 pounds with a BMI of 29.16. He had no history of trauma and no other significant past medical, surgical or psychiatric history. He was taking 7.5 mg meloxicam once a day, and methocarbomol 500 mg and acetaminophen on an as-needed basis. The patient did not meet any of the prognostic variables for serious pathology such as age <20 or >45 with no precipitating event, night pain, pain that causes the patient to be constantly moving or curled up in the sitting position, or constitutional symptoms (fatigue, nausea, diarrhea, fever). He had difficulty getting to sleep due to finding a comfortable position, but was not waking up due to pain. The patient’s goals were to stay in the military and deploy to Southeast Asia in one month with his unit. 

Physical examination

**Figure 1:**
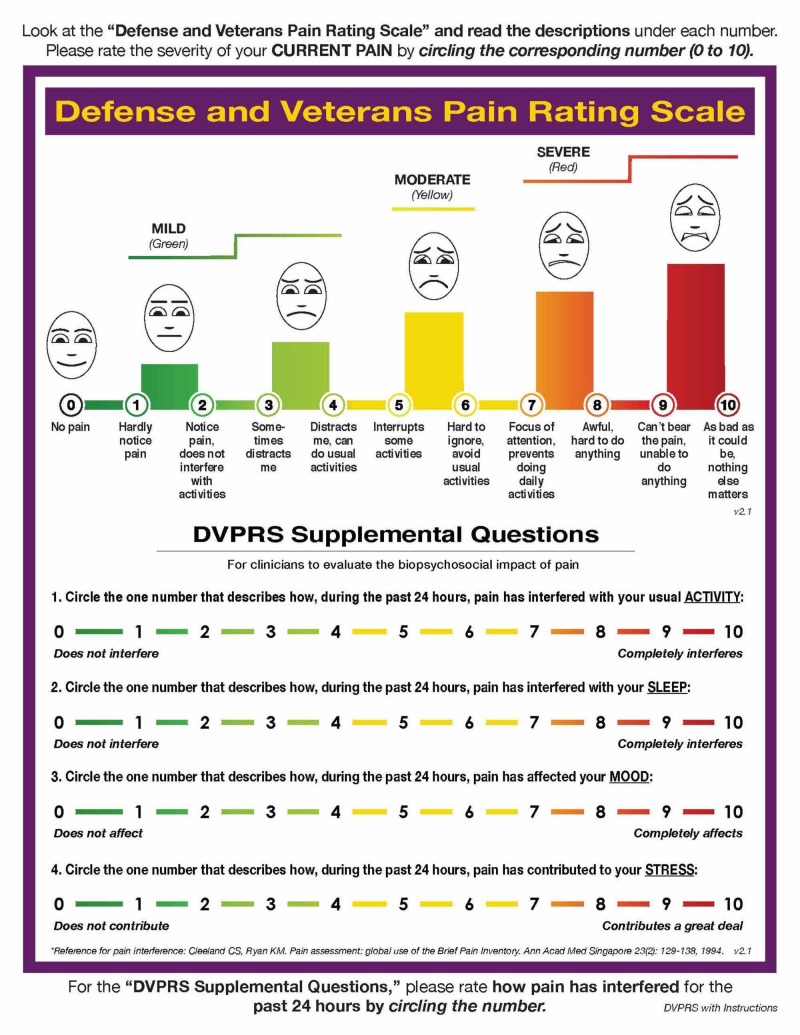
Defence and Veterans Pain Rating Scale (DVPRS)

Physical evaluation at the IPMC revealed that the patient’s pain was localized to the left posterior superior iliac spine (PSIS), the left piriformis, and along the insertion of the thoracolumbar fascia at the sacral base. The patient completed a Defense and Veterans Pain Rating Scale (DVPRS) (Figure 1) [8] with his pain level rated at 8/10. The subsets of the pain supplemental questions for activity, sleep, mood, and stress were rated at the initial encounter as 9/10, 7/10, 7/10, and 9/10, respectively, and were assessed at each follow-on visit (Table 1). The patient did not report any pain along the lumbar spine. Pain down the left posterior leg was not present at the time of the initial evaluation. A neurological clearing examination of L1-S1 revealed no dermatomal sensation changes or myotomal weakness in his bilateral lower extremities. The straight leg raise test was negative bilaterally. The patient’s lumbar range of motion (ROM) was assessed in standing using an inclinometer. He demonstrated full lumbar extension and side bending. At 30 degrees of lumbar flexion, he reported an increase in pain at the left PSIS. Hip ROM was assessed in supine with the hip flexed to 90 degrees using a goniometer. There was a 10 degree decrease in internal rotation on the left side compared to the right. Posterior anterior pressure applied manually to the spinous processes of L1 through L5 into the end range of joint motion did not change his pain. 

**Table 1:** Defence and Veterans Pain Rating Scale (DVPRS) Per Visit

	First visit	Second visit	Third visit	Fourth visit	Fifth visit	Sixth visit	Seventh visit	Final visit
Current pain level	8	8	5	4	2	2	1	0
Pain with activity	9	9	5	4	2	2	1	0
Pain affecting sleep	7	7	5	3	1	1	1	0
Pain affecting mood	7	7	6	4	1	1	1	0
Pain contributed to stress	9	9	7	4	1	1	1	0

Pain provocation tests

To determine if the patient met the SIJ clinical prediction rule (CPR) for identifying joint dysfunction [1], the following pain provocation tests were used in this study: (1) thigh thrust, (2) Gaenslen’s test, (3) sacral thrust, (4) distraction test, and (5) compression test. For the thigh thrust, the patient is supine with hip and knee flexed. The examiner cups the sacrum with one hand and applies force axially through the knee providing a shear force to the SIJ. During the Gaenslen’s test, the patient lies supine with one leg over the side of the table and the opposite knee pulled towards the chest. The examiner applies pressure against the knee towards the chest and through the thigh towards the floor. The sacral thrust involves the examiner applying a downward force to the sacrum with the patient prone. In the distraction test, the patient lies supine while the examiner applies a posterior force to bilateral anterior superior iliac spines at the same time. Finally, during the compression test, the patient lies on his side and the examiner applies a downward pressure on the upper iliac crest. Each of these tests are considered positive if pain is reproduced. Tests (1) through (3) were positive while (4) and (5) were negative. When three or more tests in this CPR are positive, the sensitivity is 0.91 and specificity is 0.78 with a positive and negative likelihood ratio of 4.5 and 4.0, respectively [1].

Motion palpation tests

In standing, inspection and palpation revealed asymmetry in the patient’s pelvic landmarks with his posterior superior iliac spine (PSIS) and iliac crest elevated on the left side as compared to the right. The motion palpation tests utilized were as follows: (1) forward flexion test, (2) Gillet’s test, and (3) the supine to sit test. In the forward flexion test, the PSIS’s are assessed for motion as the patient bends forward from a standing position. The response is positive if the PSIS moves first and/or higher on the painful side. In the Gillet’s test, the PSIS is palpated as well as the S2 spinous process while the patient is standing. The patient raises his knee towards his chest. If the PSIS on the painful side remains at the same level or raises, the test is positive. The supine to sit test allows the examiner to assess leg length while the patient slowly moves from a supine position to a long sit position. The movement of the medial malleoli is compared with a change in symmetry indicating a functional leg length discrepancy. In this case, all of the tests were positive on the left side and negative on the right side [9].

Strength and flexibility assessment

During the initial evaluation, the patient’s flexibility was also assessed. The patient presented with tightness of the iliopsoas, piriformis, and hamstrings on the involved side. Gluteus medius weakness was assessed in side-lying with manual muscle testing. The patient’s hip abduction was weaker on the left side compared to the right.

Intervention

**Figure 2:**
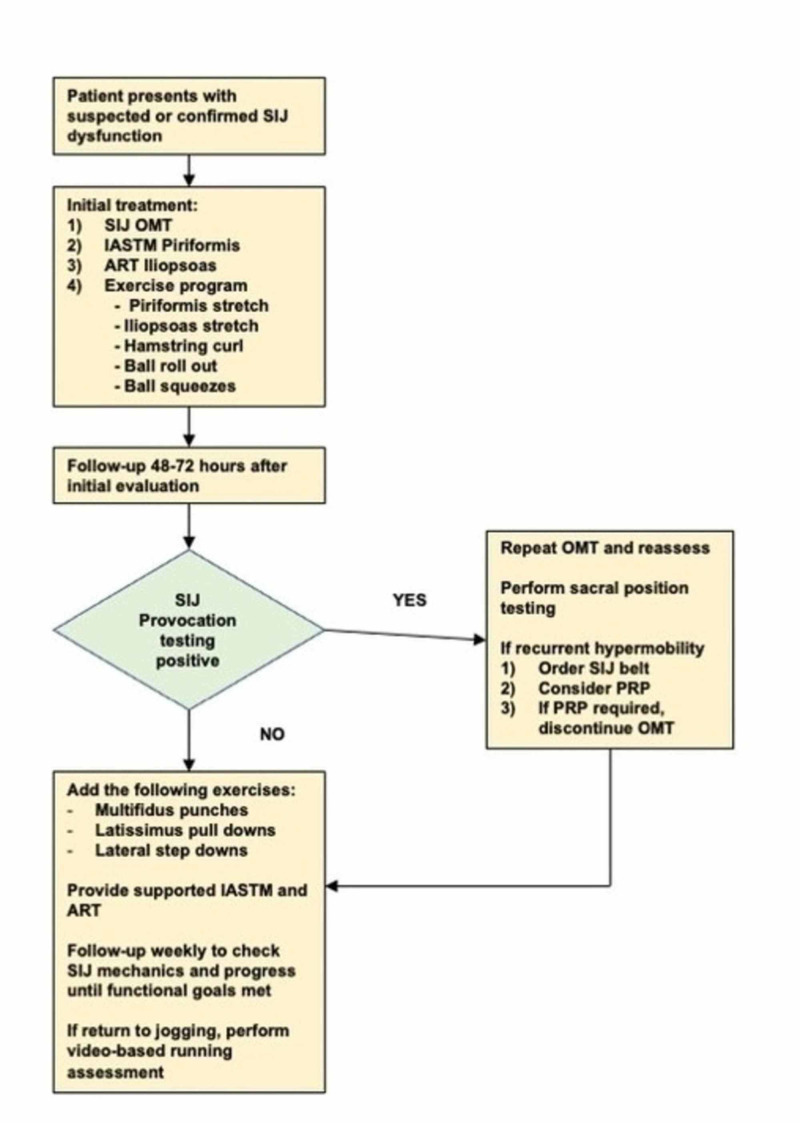
Tripler Army Medical Center Interdisciplinary Pain Management Clinic Management Algorithm This evaluation and management algorithm is provided to patients diagnosed with SIJ dysfunction or started approximately three to seven days after completion of an SIJ corticosteroid injection (CSI) or Piriformis injection (Botox or CSI). IASTM = Instrument Assisted Soft Tissue Mobilization (i.e. cupping, pneumatic/compressive massager, guasha, Graston device, etc.). ART = Active Release Technique. OMT = Osteopathic Manipulation Technique PRP = Platelet-rich Plasma

**Figure 3:**
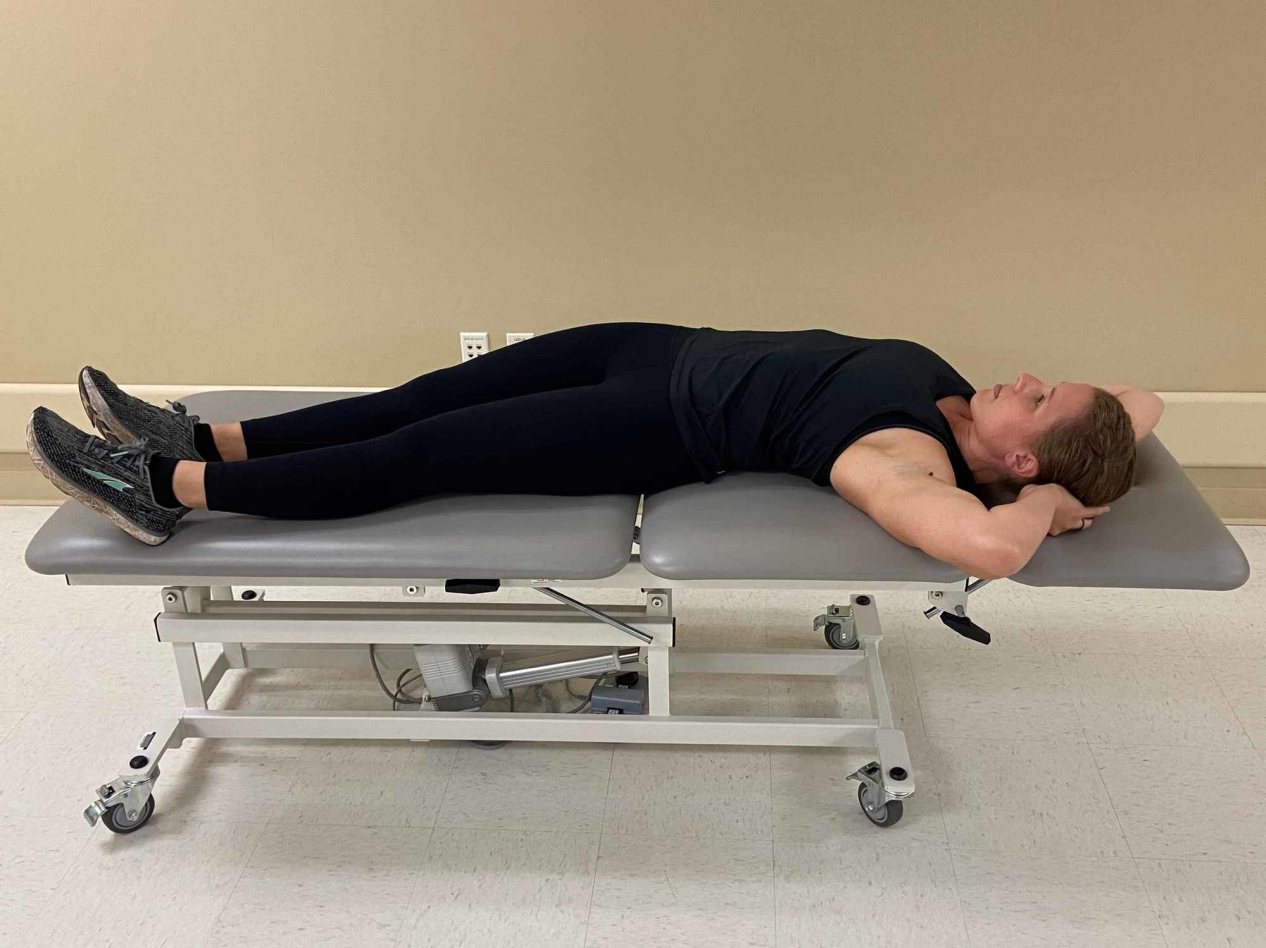
Osteopathic Manipulation Technique – Patient Positioning The patient is placed in supine with hands clasped behind their neck. The examiner passively moves both legs and torso towards the left side (Photograph: Scozzafava, AM. Osteopathic Manipulation Technique - Patient Positioning. Reproduced by permission of author; 2020).

**Figure 4:**
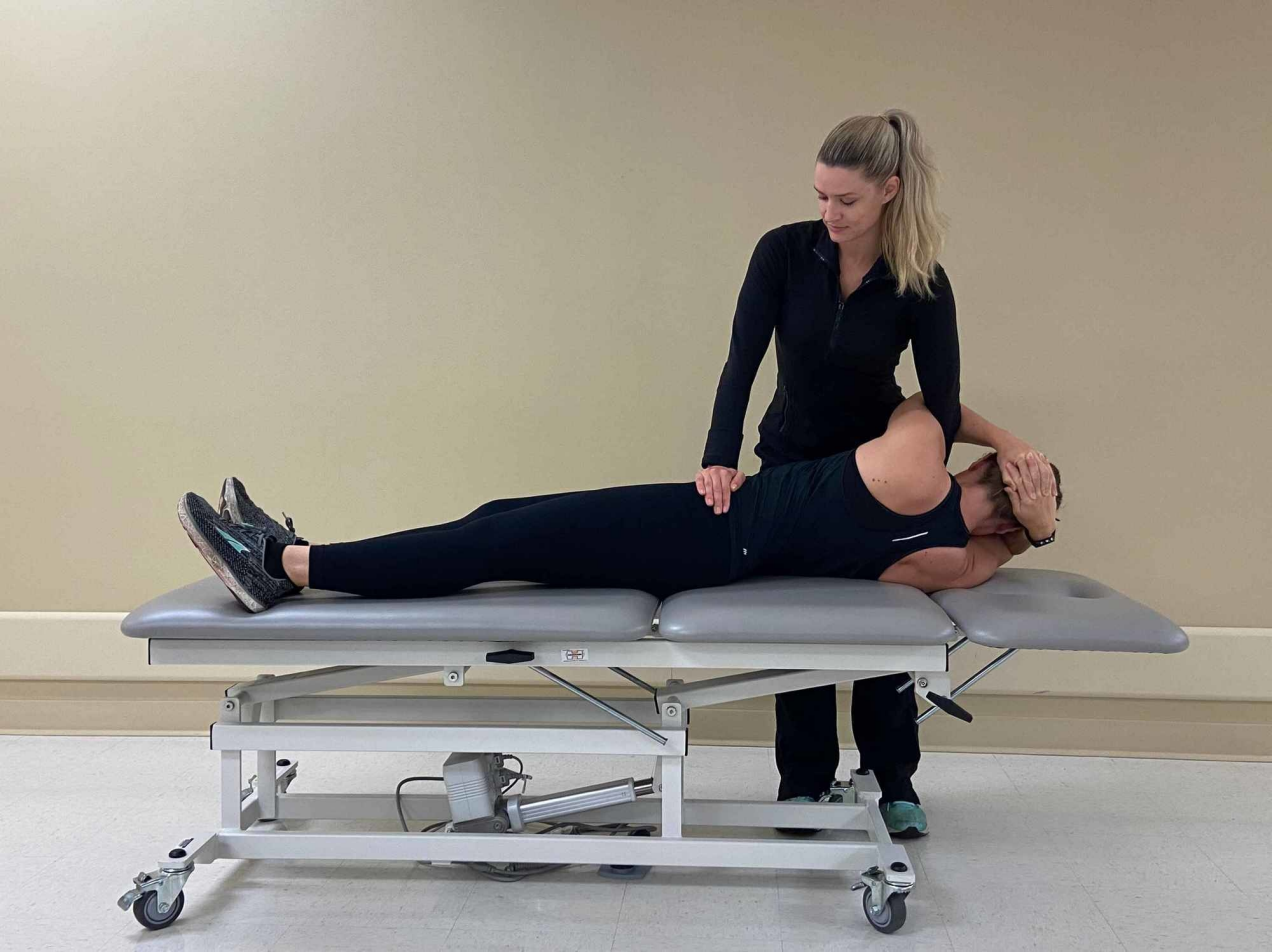
Osteopathic Manipulation Technique – Thrust Maneuver The torso is then rotated to the right fully, thereby locking the spine. The examiner’s caudal arm is placed through the patient’s arms to stabilize the torso while the other hand grips the left iliac bone. While the patient exhales, the examiner simultaneously rotates the torso farther while imparting a high velocity, low amplitude force through the ilium (Photograph: Scozzafava, AM. Osteopathic Manipulation Technique - Thrust Maneuver. Reproduced by permission of author; 2020).

The patient was treated according to an algorithm developed at the IPMC (Figure 2). Upon initial treatment, he underwent a common osteopathic manipulation technique (OMT) directed at the left ilium to address the SIJ dysfunction (Figures 3, 4) [10]. This maneuver resulted in immediate improvements in lumbar flexion to 50 degrees as well as negative provocation testing (i.e. thigh thrust and sacral thrust) and motion palpation tests (i.e. forward flexion test and Gillet’s test).

 

To identify the impact of piriformis tightness upon the patient’s pain presentation and the effect of stretching upon achieving treatment success, instrument assisted deep tissue mobilization was performed using the VibraCussor® (IMPAC Inc., Salem, OR) directed at the piriformis for ten minutes. This treatment was followed by manual passive stretching of the piriformis in supine. Following treatment, the patient demonstrated full lumbar motion and hip internal rotation without pain. The patient was started on a home exercise program consisting of piriformis stretching and lumbopelvic stabilization exercises directed at improving muscle endurance of the abdominals, hamstrings, and hip adductors. The patient was asked to follow up three days later.

Upon the first follow-up, the patient reported that the pain relief experienced at the first visit lasted two days, but that repeated forward bending during a routine task of daily living, specifically making dinner, resulted in a return of his baseline pain levels. Examination revealed pelvis asymmetry and piriformis tightness similar to the initial evaluation. The treatment as described in the algorithm was repeated, and he was instructed to follow up two days later.

By the third visit, there was a precipitous reduction in pain during activity and sleep. The same treatment performed at the previous two visits was repeated. The patient again reported an immediate reduction in pain with full lumbar motion. By the fourth visit, assessment of the pelvic landmarks was symmetrical and motion testing was negative. The patient was encouraged to start squatting and take a slow jog to assess progress towards goal achievement. At reassessment on the fifth visit, he continued to report reductions in pain across all subsets of the DVPRS. 

At the sixth and seventh visits, directional cupping was applied to address thoracolumbar fascia tightness. Active release followed by manual stretching to the iliopsoas was provided to improve flexibility. The directional cupping technique involved the application of five plastic cups to the area of the sacral base and lower lumbar paraspinals. Once the suction was induced, the cups were manually moved up/down and back/forth for a period of five minutes. For the active release, pressure is applied to the iliacus muscle against the pelvic rim while the patient actively extends his leg from hip flexion to neutral in supine. This technique is repeated several times until the muscle relaxes and there is less resistance felt during passive stretching.

Upon the eighth and final visit, the patient reported no pain across all DVPRS subsets. The patient reported squatting 135 pounds to a depth and intensity greater than his pre-injury level. He was able to perform all physical activities required for his physical fitness test without pain. He was subsequently discharged from the IPMC and deployed the following week.

## Discussion

Conservative management of patients with SIJ dysfunction can be provided via a multi-modal rehabilitation program directed at re-establishing the mechanical relationship of the SIJ and surrounding joints, supporting tissues, and potential biomechanical faults remote to the SIJ [11]. This case study demonstrates the successful application of an evaluation and management algorithm that combines manual/manipulative therapy with exercise and tissue mobilization in a patient with low back pain due to SIJ dysfunction.

The combination of exercise with manipulative therapy was chosen due to the time constraints of the patient given his upcoming deployment to Southeast Asia in four weeks. The SIJ OMT was selected as it has been shown to be very effective for pain management in the short term [10]. Evidence suggests that patients who receive an exercise program alone, manipulative therapy alone, or a combination of both, significantly improve compared to their baseline at 12 weeks for objective outcomes and at 24 weeks for subjective outcomes. However, patients receiving manipulative therapy with or without exercise, receive a more acute benefit as compared to exercise alone [12]. In patients with sciatica, Visser and colleagues demonstrated better results in pain and functional outcomes with two sessions of manipulative therapy to the SIJ over a period of two weeks as compared to a six-week program focusing on stretching and lumbar and pelvic floor strengthening [13]. Exercises play an important role in long term benefit [14]; therefore, both treatment modalities were utilized in this rehabilitation program.

Optimization of muscle activation patterns is critical to SIJ stability [6]. In the applied treatment algorithm, the initial lumbopelvic stabilization program consisted of strengthening the hip adductors, hamstrings, and abdominals in pain-free positions. The abdominals (i.e. transversus abdominus, rectus abdominus, and obliques) are necessary to achieve SIJ stability, especially when vertical stabilization is decreased due to injury [15]. 

The prescribed stretching program was designed to address piriformis and iliopsoas tightness commonly noted in patients with SIJ dysfunction [16]. Piriformis tightness limits hip motion and can result in irritation of the sciatic nerve. In the presence of altered abdominal function, the iliacus increases anterior ilial rotation and magnifies anterior abdominal forces upon the spine and SIJ [6].

Thoracolumbar fascia (TLF) mobilization was provided near the end of the treatment course. The TLF plays a role in stabilizing the SIJ, posture, and reducing compressive strain upon the spine during activation of the extensors [17]. The posterior fascial layer assists in transferring force between the spine, pelvis, and lower extremities while the aponeurotic component links the abdominals to the lumbar paraspinals [18]. Connective tissue pathology results in decreased shear strain in patients with low back pain [19]. The TLF can be treated with directional (moving) cupping, performing range of motion exercises with the cups applied over the fascia, and/or foam rolling. In this case, these techniques provided immediate improvement in pain and motion as well as complemented the manual therapy and exercise program.

This case study does have several limitations. The result may not be generalizable to all patients with SIJ pain. This patient likely did not have a degenerative sacroiliitis or inflammatory condition which may be more common in older patients with sacroiliac joint pain. Although our treatment algorithm is similar for this subset of patients, treatment success with end-stage osteoarthritis of the sacroiliac joint may be less successful. Our subject was young, physically fit, and duty motivated to return to work. These factors are not congruent with the prototypical chronic low back pain patient who is more likely overweight, deconditioned, and presents with other medical comorbidities. Finally, since this patient deployed and is not available for follow-up, it is difficult to gauge the long-term benefit from this algorithmic treatment program.

## Conclusions

The case presentation and treatment algorithm presented herein represents our standard approach to patients with chronic low back pain due to SIJ dysfunction. Patients present to our IPMC after failure of rest, conservative care, and conventional physical therapy evaluation and management. This patient population is challenging in that common interventional pain procedures often do not provide clinically significant treatment success and functional improvements in isolation. We have found that by instituting the described interdisciplinary treatment algorithm, we have successfully not only managed the patients’ pain but in many cases corrected the underlying physical or mechanical dysfunction. Further case-controlled studies should be used to validate this algorithmic approach to SIJ dysfunction.
